# Metabolomics analyses identify platelet activating factors and heme breakdown products as Lassa fever biomarkers

**DOI:** 10.1371/journal.pntd.0005943

**Published:** 2017-09-18

**Authors:** Trevor V. Gale, Timothy M. Horton, Donald S. Grant, Robert F. Garry

**Affiliations:** 1 Department of Microbiology and Immunology, Tulane University, New Orleans, Louisiana, United States of America; 2 Viral Hemorrhagic Fever Program, Kenema Government Hospital, Kenema, Sierra Leone; 3 Ministry of Health and Sanitation, Freetown, Sierra Leone; 4 Zalgen Labs, LLC, Germantown, Maryland, United States of America; 5 Tulane Center of Excellence, Global Viral Network, New Orleans, Louisiana, United States of America; University of Texas Medical Branch, UNITED STATES

## Abstract

Lassa fever afflicts tens of thousands of people in West Africa annually. The rapid progression of patients from febrile illness to fulminant syndrome and death provides incentive for development of clinical prognostic markers that can guide case management. The small molecule profile of serum from febrile patients triaged to the Viral Hemorrhagic Fever Ward at Kenema Government Hospital in Sierra Leone was assessed using untargeted Ultra High Performance Liquid Chromatography Mass Spectrometry. Physiological dysregulation resulting from Lassa virus (LASV) infection occurs at the small molecule level. Effects of LASV infection on pathways mediating blood coagulation, and lipid, amino acid, nucleic acid metabolism are manifest in changes in the levels of numerous metabolites in the circulation. Several compounds, including platelet activating factor (PAF), PAF-like molecules and products of heme breakdown emerged as candidates that may prove useful in diagnostic assays to inform better care of Lassa fever patients.

## Author summary

Lassa fever afflicts tens of thousands of people in West Africa each year. The disease progresses rapidly, but there are no tests available to determine which patients are at high risk for dying. We measured the levels of small molecules in the blood of febrile patients with and without infection by LASV that presented to Kenema Government Hospital in Sierra Leone using Ultra High Performance Liquid Chromatography Mass Spectrometry (LCMS), which identifies compounds based on their precise mass. Computational analyses were used to identify compounds that differed in patients with an acute LASV infection, patients with evidence of prior exposure to LASV and patients with fever, but who did not have evidence of exposure to LASV. Several serum metabolites, including factors that are involved in blood clotting and breakdown products of heme, were identified that may prove useful in diagnostic assays that will inform better care of Lassa fever patients or development of therapeutic interventions.

## Introduction

Lassa virus (LASV), an Old World arenavirus, is the etiological agent of Lassa fever [1]. Lassa fever is endemic to West Africa, with tens of thousands of cases or more estimated to occur annually [2]. The case fatality rate (CFR) in acutely ill Lassa fever patients presenting while viremic was 29–31% in Nigeria [3,4] and 69% in Sierra Leone [5]. During a recent surge of Lassa fever cases in Nigeria the CFRs were >50% [6]. Women who are pregnant develop severe disease with increased frequency and have a Lassa fever CFR as high as 90%, with fetal death, miscarriage or spontaneous abortion occurring in nearly all cases [7,8]. Recent cases in Togo, Benin and areas of Nigeria that rarely have Lassa fever [6,9,10], coupled with serological studies in Mali [11–14], suggest that efforts to improve Lassa fever surveillance should continue [15]. Lassa fever ranks among the most common of the viral hemorrhagic fevers that are imported from Africa [16–20]. Recently, the first case of Lassa fever contracted outside Africa was reported in Germany [21]. There is no approved Lassa fever vaccine, and the only available treatment, ribavirin, is effective only during early infection [5,22,23]. Management of Lassa fever principally involves supportive therapy such as fluid replenishment for dehydration [5].

Lassa fever presents in its early stages as a febrile illness indiscernible from multiple diseases of infectious etiology that are common in West Africa, such as malaria, typhoid, leptospirosis, influenza and various arbovirus-induced illnesses [24]. After the brief nondescript prodrome, progression to fulminant disease occurs rapidly [25]. Lassa fever diagnostics include enzyme linked immunosorbant assays (ELISA) detecting LASV antigen (Ag), human anti-LASV IgG and IgM, a rapid lateral flow immunoassay [5], and polymerase chain reaction based assays detecting viral genomic RNA [26]. Diagnostics can provide the serostatus of patients presenting with a febrile illness and offer a quantitative view of antibody responses. However, the presence of anti-LASV IgM lacks utility as a reliable marker of recent LASV infection [27], and IgM persistence can be confounding as a strategy to monitor disease progression [28]. High viremia is an indicator for poor outcome in Lassa fever [28]. At present, few other markers exist that accurately inform clinical management of Lassa fever patients or patients that survive the acute illness. Increases in serum markers of hepatic damage, particularly aspartate aminotransferase (AST), increase during the acute stage of Lassa fever and levels correlate with fatal outcome in Lassa fever [28–30]. Likewise, the serum levels of cytokines interleukin 6, 8, and 10 (IL-6, IL-8, IL-10), macrophage inflammatory protein 1 alpha & beta (MIP-1α/β), and interferons-alpha/gamma (INF- α/γ) are altered during the course of the disease [28,31–35]. However, subjects that survive also display very high serum levels of liver enzymes or various cytokines, which limits the prognostic value of these markers.

Lassa virus exhibits tropism for circulating leukocytes, and there is evidence to suggest LASV infection results in endothelial dyregulation [36–38] that results in loss of intravascular volume [39]. These circulatory pathologies suggest that the serum of a Lassa fever patient may be an informative, clinically relevant medium to monitor perturbations to homeostasis and may have utility in tracking the trajectory of disease. Furthermore, analysis of Lassa fever at the small molecule level may reveal intermediates of cellular pathways disrupted by LASV replication, suggesting pathogenic mechanisms that the virus utilizes and identifying markers for prognostic diagnostics and potential targets for intervention strategies. Herein, we report findings from a Liquid Chromatography Mass Spectrometry (LCMS) serum metabolomics investigation of a heterogeneous clinical population presenting with febrile illness and triaged to the Lassa Fever Ward at Kenema Government Hospital (KGH) in Sierra Leone.

## Methods

### Human subjects

The Tulane University Institutional Review Board and the Sierra Leone Ethics and Scientific Research Committee approved this project. Patients were referred to the KGH Viral Hemorrhagic Fever Ward from the hospital’s general ward or from regional health centers on the basis of suspicion of Lassa fever. Patients who met the case definition of Lassa fever as defined by Khan et al. [40] were admitted and cared for by the ward’s trained staff. All adult subjects provided written informed consent for publication of their case details. A parent or guardian of child participants provided written informed consent on their behalf.

Small blood volumes (approximately five ml) for serum separation were collected from patients presenting to KGH with febrile illnesses that met preclinical criteria of suspected Lassa fever for diagnostic purposes. Patient samples received a coded designation and were collected in serum vacutainer tubes. Blood samples were allowed to coagulate for 20 minutes at room temperature. Serum was separated from coagulated blood by centrifugation (200 x g, 20 minutes at room temperature). For consented subjects for which there was excess serum not needed for clinical evaluations, aliquots of the serum fraction were stored in cryovials at -20°C prior to processing for metabolite analysis.

### Lassa fever recombinant immunoassays

A lateral flow immunoassay requiring only a drop of blood obtained with a safety lancet and capable of detecting LASV antigenemia within 15 minutes was utilized to triage cases for possible LASV infection [28,35]. Serum from subjects was subsequently tested using recombinant antigen-based Lassa fever antigen-, IgM- and IgG-capture enzyme-linked immunosorbent assays (ELISA) [24]. Limits of detection and quantitation of the ELISA were based on the upper 95th percentile obtained with a panel of sera from U.S. and Sierra Leonean donors lacking detectable LASV antigens or immunoglobulin M or G (IgM, and IgG) antibodies to LASV recombinant proteins.

### Serum processing for metabolomics analyses

All sera collected at the Lassa Fever Ward, KGH is treated as if it contains replication-competent LASV. Serum samples were prepared via a validated metabolomics preparation method utilizing ice-cold methanol for extraction [41,42]. Separated serum samples were depleted of protein by addition to one part sera (100 μL) of 4 parts ice-cold methanol (400 μL), the mixture was vortexed vigorously for 10 seconds, and incubated 1 hour at -20°C followed by centrifugation at 14,000 x g, 15 minutes, 4°C. The supernatant was collected and transferred to a new, sterile vial and dried under vacuum. The resultant small-molecule containing pellets were stored in desiccated, sealed containers and shipped to Tulane University where they were gamma-irradiated. Small molecule containing pellets were dissolved in a solution of 95:5 water:acetonitrile transferred to autosampler vials, and held at -20°C or 4°C immediately prior to analysis [43]. All reagents utilized were HPLC grade.

### Liquid Chromatography Mass Spectrometry

LCMS methods was optimized based upon a meta-sample consisting of an equal-volume mix of all 50 samples. Detection of metabolites was performed via HPLC separation with ESI-MS (electrospray mass spectrometry) detection. HLPC was performed with an aqueous normal-phase, hydrophilic interaction chromatography (ANP/HILIC) HPLC column: a Cogent Diamond Hydride Type-C column with 4 μm particles and dimensions of 150 mm length and 2.1 mm diameter was used with an Agilent 1290 HPLC system (Agilent Technologies, Santa Clara, CA). Two identical Diamond Hydride columns were connected in series to obtain better separations. The column were maintained at 60°C with a flow rate of 900 μL/min. Chromatography was as follows: solvent consisted of H_2_0 with 0.1% (v/v) formic acid for channel “A” and acetonitrile with 0.1% formic acid for channel “B”. Following column equilibration at 98% B, the sample was injected via autosampler, and the column was flushed for 2.0 min to waste. From 2.0 min to 14.5 min, the gradient was linearly ramped from 98% to 65% B. From 14.5 min to 16.0 min, the gradient was ramped from 65% to 25% B. From 14.5 to 18.0 min the column was held at 25% B, and from 18.0 to 18.2 minutes the gradient was ramped from 25% to 98% B. From 18.2 to 20.0 minutes the column was re-equilibrated with 98% B. An Agilent 6538 Q-TOF with dual-ESI source mass spectrometer was used for all analyses. Resolution was approximately 20,000 and accuracy was 1 ppm. Source parameters were: drying gas 12 L/min, nebulizer 60 psi, capillary voltage 3500V, capillary exit 100V. Spectra were collected in positive mode from 50 to 1700 *m/z* at a rate of 1 Hz.

### Data analysis and visualization

Raw spectral data in .d format where uploaded to XCMS Online (Version 1.0.42) and processed as pairwise comparisons using parameters optimized for data acquired with UPLC on an Agilent 6538 MS [44]. Data has been deposited in the XCMS Public archive (https://xcmsonline.scripps.edu/landing_page.php?pgcontent=mainPage) under the identifier Lassa_Serum_PLOS_NTD.

### Statistics and machine learning

All statistical analyses were carried out using the R statistical software package [45]. Raw mass spectral intensity values and a unique identifier for specific spectral features were extrapolated from XMCS output and compiled into .csv files for machine learning analysis with predefined outcome. Machine learning algorithms built into the R package were utilized with outputs quantifying the sensitivity, specificity, accuracy, and/or receiver operating character computed depending on diagnostic (binary) or prognostic (multi-outcome) analysis. The Random Forest algorithm was employed for all analyses reported. The Random Forest algorithm was set to select features through permutation of the data set yielding the strongest indicators of the input features. The datasets where run with 10-fold cross validation ensuring that ranked output features where selected on importance after predefined, multiple rounds of random training and testing. FacoMineR was utilized for a Principle Components Analyses [46].

## Results

### Demographics of patients presenting to the Kenema Government Hospital Viral Hemorrhagic Fever Ward

**Fig 1:**
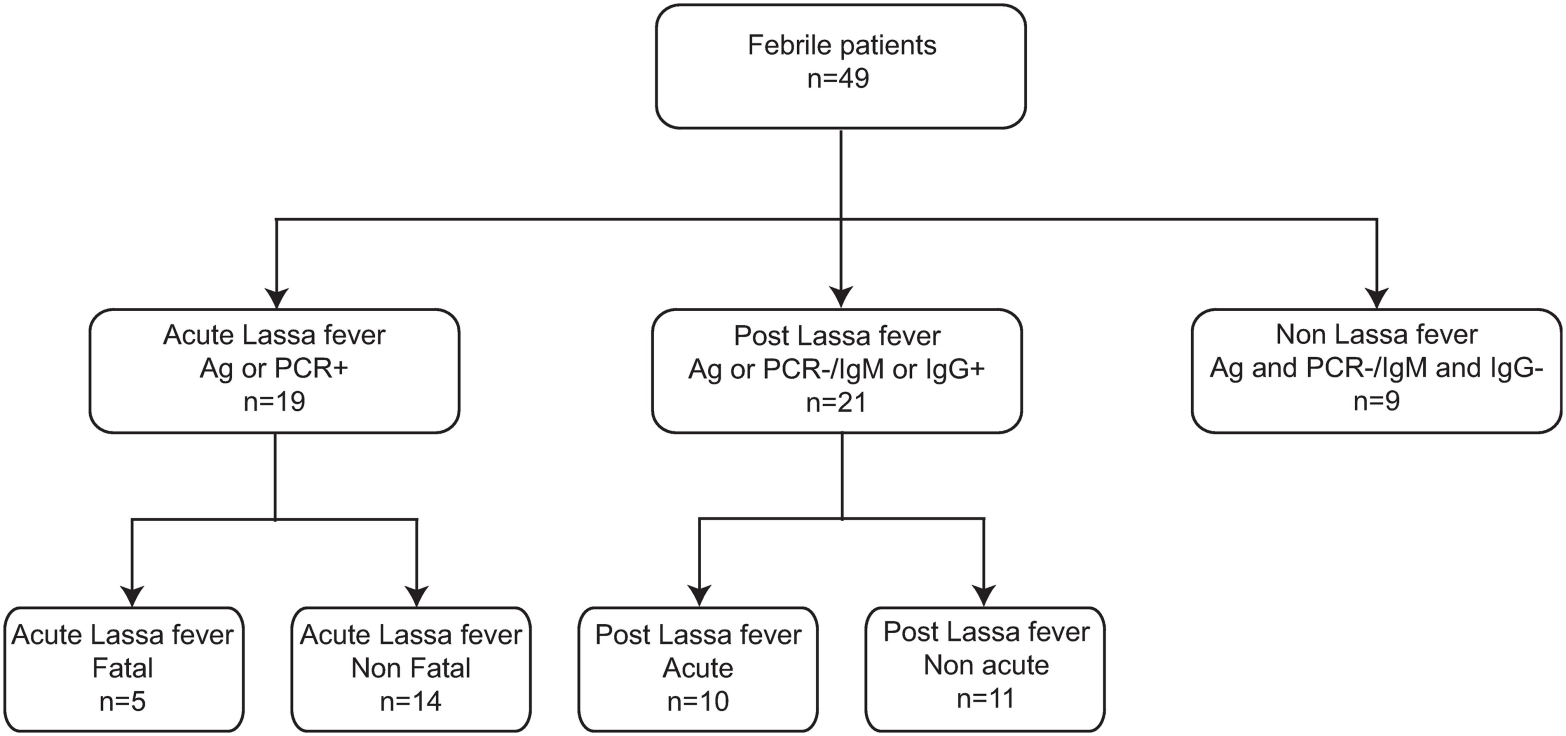
Serostatus of febrile patients presenting to the Kenema Government Hospital Viral Hemorrhagic Fever Ward. Subjects meeting the case definition for possible viral hemorrhagic fever were tested for the presence of LASV in the blood by RT-PCR or antigen-capture ELISA. The presence of anti-LASV IgM or IgG was assessed by recombinant antigen ELISA.

A panel of 49 serum samples from patients presenting to Kenema Government Hospital (KGH) in Sierra Leone and triaged to the Viral Hemorrhagic Fever Ward (LFW) with febrile illnesses was analyzed by LCMS. These subjects presented with varying serological status and are representative of the spectrum of illnesses during Lassa fever from acute disease to convalescence (Fig 1). Serum samples where drawn upon admittance. Diagnostic tests for patients were performed the same day the sample was drawn. Twenty subjects tested positive for the presence of LASV in their blood by either antigen-capture ELISA or RT-PCR and were considered to have acute Lassa fever. Five patients died and were classified as having fatal Lassa fever (FL). 15 of these patients survived and were classified as having non-fatal Lassa fever (NFL). 21 subjects without measurable LASV in there blood tested positive for the presence of anti-LASV Immunoglobulin M (IgM) and/or anti-Lassa IgG by ELISA, and were considered to have survived infection with LASV. This group was subdivided into post-Lassa fever patients that were acutely ill (PLAFI, n = 9) or those whose illness was non-acute and presented after an extended period of illness (PLNAFI, n = 11). Nine patients presenting with a febrile illness, but testing negative for the presence of LASV and either anti-Lassa IgM or IgG and were classified as non-Lassa febrile illness (NLF) controls.

There were 28 female and 21 male patient sera screened (57% female) with gender information not available for one patient (S1 Table). The mean age was 26.0±13.9 years with age information not available for one patient. 7 patients died (14% total sample group) with a mean terminal time point (time in days since the onset of symptoms) of 11.4±5.1 days (with time since symptom onset not available for two patients). The mean age for patients who died was 24.6 ±9.7 years.

The antiviral drug ribavirin has been reported have some efficacy in the treatment of Lassa fever, particularly if treatment is begun early during the course of the illness. In this cohort eleven patients with Lassa virus viremia at the time of admission, as well as three IgM positive patients received ribavirin. Spectral features of protonated, sodiated, and potassiated (*m/z* = 245.0852, 267.0775, & 283.0408; rt = 4.30, 4.32, 4.34, respectively) ribavirin adducts where detected in these samples. Three of the 11 (21%) ribavirin treated acute Lassa fever patients died.

### The serum metabolome is dynamic following LASV infection

**Fig 2:**
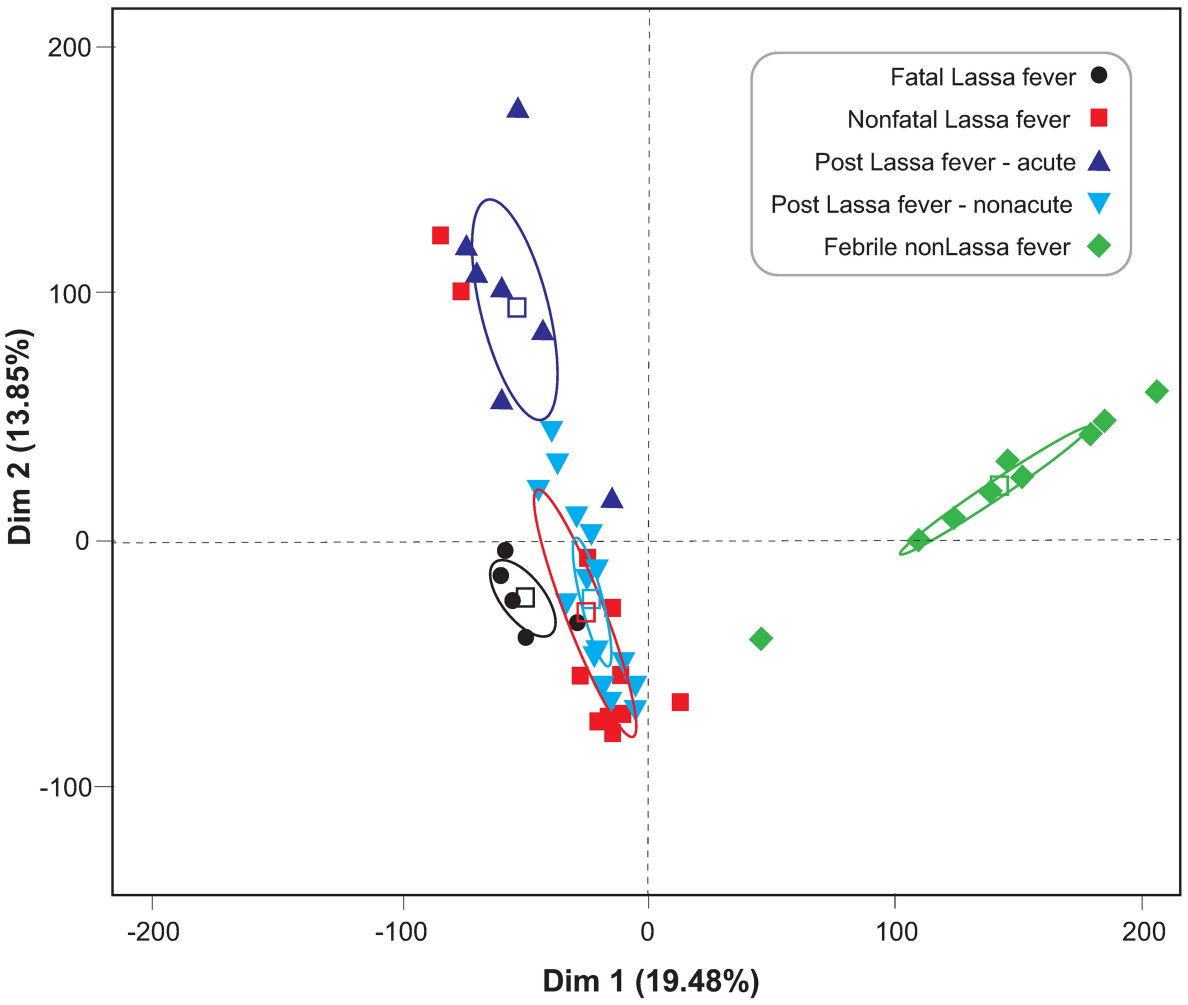
Principal component analysis of serum metabolites in subjects with different Lassa virus serostatus. Spectral values extracted from post-XCMS data for each sample where grouped by serostatus and time since symptom onset and PCA computed in R using the FactoMineR package. Fatal Lassa fever (black circles), Non-fatal Lassa fever (red squares). Post Lassa fever—acute illness (dark blue triangles). Post-Lassa fever—non-acute illness (light blue triangles). Febrile non-Lassa illness (green diamonds).

Individual samples produced between 3100 and 6900 different spectral features. LCMS analyses allowed for the putative identification of small molecules in serum from the cohort of febrile patients presenting to KGH. Principle Components Analysis (PCA) of these features indicated that Lassa fever patients with different outcomes and patients at various stages during and after LASV infection segregated according to their serum small molecule profiles (Fig 2). Patients with active or prior LASV infection had profiles that were distinct from febrile patients without serological evidence of current or prior LASV exposure. The small molecule profiles of patients with fatal and non-fatal Lassa fever were also distinct. The overall serum small molecule profile of patients with evidence of prior exposure to LASV and presenting to the Lassa fever Ward after an extended period of illness (post-LASV non-acute) most closely resembled the profile of nonfatal Lassa fever patients. Patients with evidence of prior exposure to LASV, but presenting with an acute illness, showed a distinct small molecule PCA profile.

### Platelet-activating factor/platelet-activating factor-like molecules are potential biomarkers in Lassa fever

**Fig 3:**
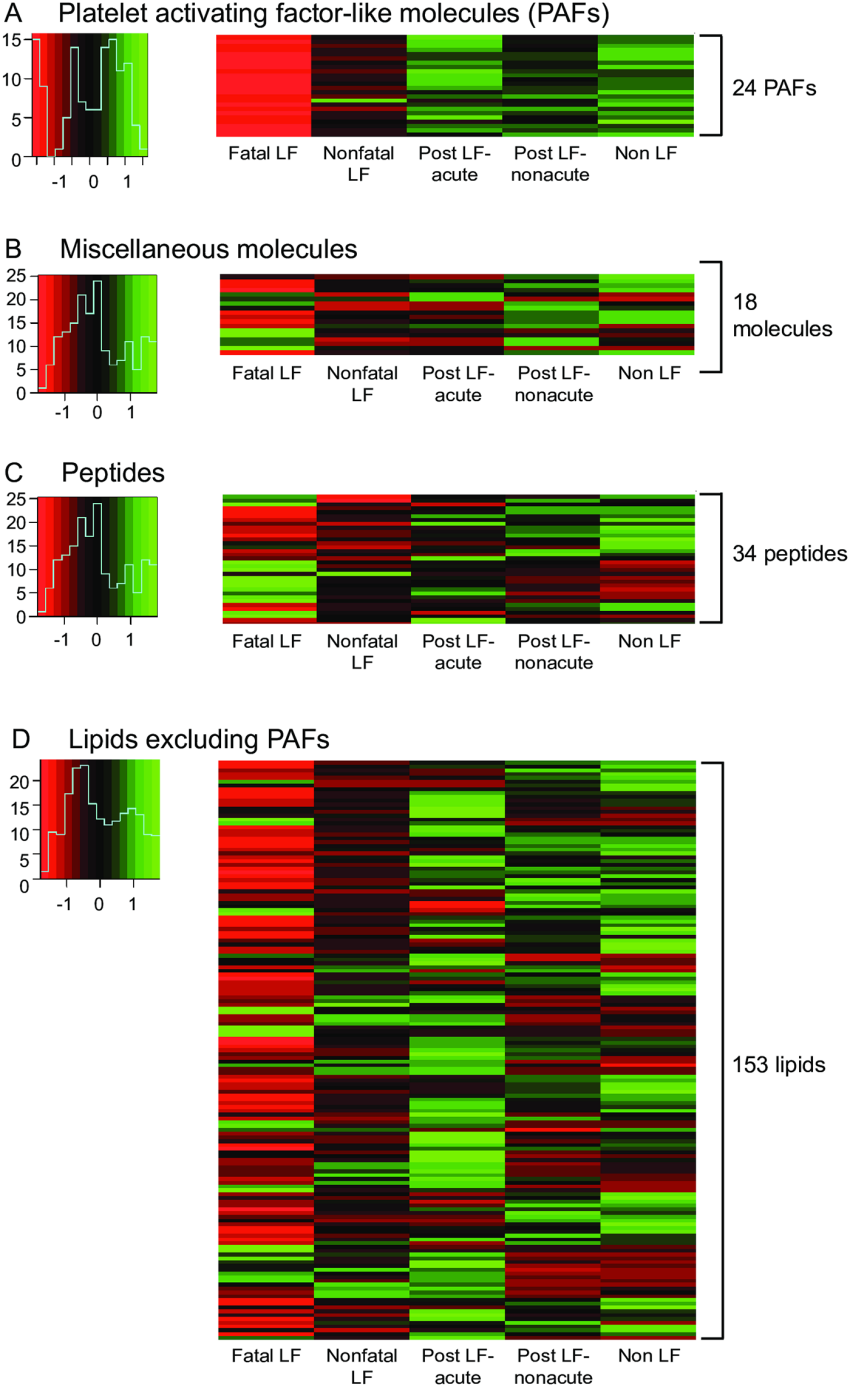
Cluster analysis of serum metabolites in subjects with different outcomes following Lassa virus infection, survivors of Lassa virus infection and febrile controls. Heat map representing levels of 24 putatively identified platelet activating factor (PAF) or PAF-like molecules, 18 miscellaneous molecules, 34 peptides and 153 lipids (no PAFs included) in subjects with different Lassa virus serostatus. Panel A: Platelet activating factor (PAF) and PAF-like molecules. PAFs and PAF-like molecules are listed in S2 Table. Panel B: Miscellaneous molecules. Metabolites are listed in S3 Table. Panel C: Peptides. Peptides are listed in S4 Table. Panel D: Lipids (no PAFs included). Lipids are listed in S5 Table.

**Fig 4:**
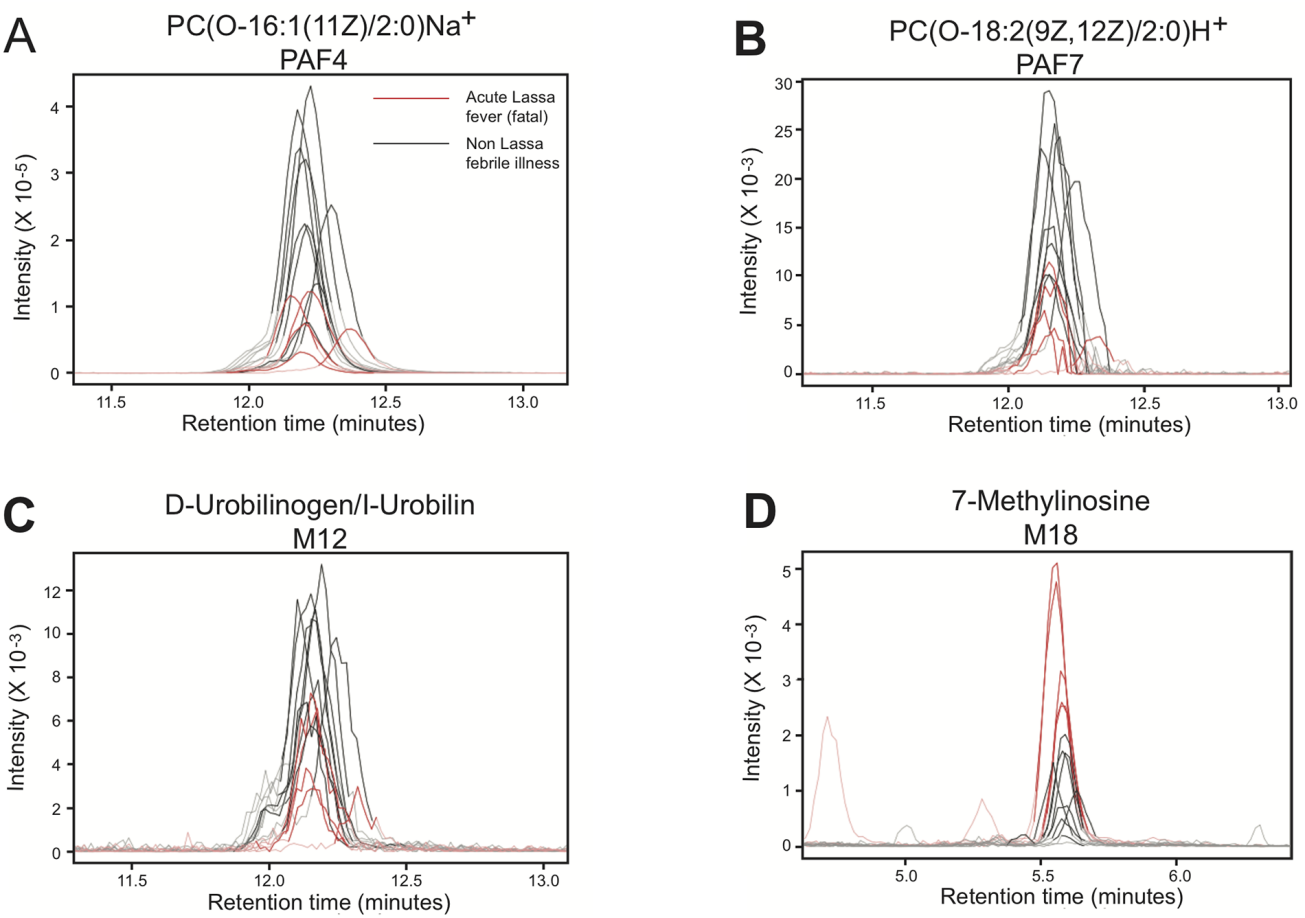
Extracted ion chromatograms of selected metabolites that differ in the serum of subjects with fatal and nonfatal Lassa fever. Intensity versus retention time plots for selected metabolites that are present in different levels in the serum of subjects with fatal Lassa fever and subjects that presented with a non-Lassa febrile illness. Panel A: PC(O-16:1(11Z)/2:0)Na^+^ PAF4. Panel B: PC(O-18:2(9Z,12Z)/2:0)H^+^ PAF7. Panel C: D-Urobilinogen/I-Urobilin M12. Panel D: 7-Methylinosine M18. Red: Acute Lassa fever—fatal. Black: non-Lassa febrile illness.

We used the LCMS data to perform cluster analysis of serum metabolites in subjects with different outcomes following Lassa virus infection, survivors of Lassa virus infection and febrile controls (Fig 3). Platelet activity is depressed during Lassa fever, particularly in terminal patients [29,47]. 24 platelet-activating factor/platelet-activating factor-like molecules were putatively identified and expressed at variable levels in the serum of febrile patients presenting to KGH (Fig 3A, S2 Table). Protonated and sodiated adducts of phosphatidylcholine, platelet-activating factor (PAF) C-16, its metabolic precursors Lyso-PAF C-16 and Arachidonoyl PAF C-16, and 9 additional PAF-like lipids were putatively identified via manual *m/z* screening. Heat maps of the levels of PAF or PAF-like species illustrate the levels from low (red), intermediate (black) to high (green) in the patient groups. The cluster analysis indicated that nearly all PAFs or PAF-like molecules were present in lower amounts in the serum of patients with fatal Lassa fever than in patients that survived the acute infection (nonfatal Lassa fever). Post-Lassa patients had higher levels of PAF or PAF-like molecules than fatal Lassa fever patients, with the subgroup of patients presenting with an acute illness displaying higher levels than the non-acute group. Non-Lassa febrile illness patients had the highest overall levels of PAF or PAF-like molecules. Extracted ion chromatograms of selected metabolites were analyzed (Fig 4). This analysis confirms the lower levels of two PAFs, PAF4 (PC(O-16:1(11Z)/2:0) H^+^, *m/z* 522.3504 and PAF 7 (PC(O-18:2(9Z,12Z)/2:0) Na^+^, 570.3463) in patients with fatal Lassa fever compared to patients with non Lassa fever (Fig 4A and 4B).

### Products of heme breakdown, nucleosides, peptides, lipids and other spectral features in the serum of patients with suspected Lassa fever

Products of hemoglobin breakdown and various nucleosides were among other spectral features that were putatively identified in the LCMS data set (Fig 3B, S3 Table). Certain of these metabolites were expressed at variable levels in the serum of febrile patients presenting to KGH. For example extracted ion chromatograms confirm that the hemoglobin breakdown products D-urobilinogen and I-urobilin sharing *m/z* 591.3195 were reduced in patients with fatal Lassa fever compared to Lassa Negative patients (Fig 4C). A spectral feature consistent with the protonated adduct of 7-methylinosine is detected with *m/z* 283.1016 (theoretical *m/z* = 283.1037) significantly elevated in the sera of Lassa fever patients who died compared to patients with a non-Lassa febrile illness and other patient groups (Fig 4D). There were several spectral features that could not be putatively identified by their precise mass, and were designated as unknown metabolites.

Lipids constituted the most abundant class of molecules assigned putative identifications in serum samples from the cohort of subjects presenting with febrile illnesses to KGH. 153 substituents of the primary lipid classes were putatively identified included fatty acids and conjugates, fatty esters, glycerophosphocholines, glycerolipids, diacyglycerols, glycerophospholipids, prenol, sterol, sphingolipids, vitamin D3 and derivative species (Fig 3D, S5 Table). Approximately half of the lipids were present in lower amounts in the serum of patients with fatal Lassa fever than in patients with non-fatal Lassa fever. Lipids as a class were generally higher in post-LASV group of patients presenting with an acute illness or in patients with a non-Lassa febrile illness than in the other patient groups.

### Machine learning to identify metabolites with diagnostic potential in Lassa fever

**Fig 5:**
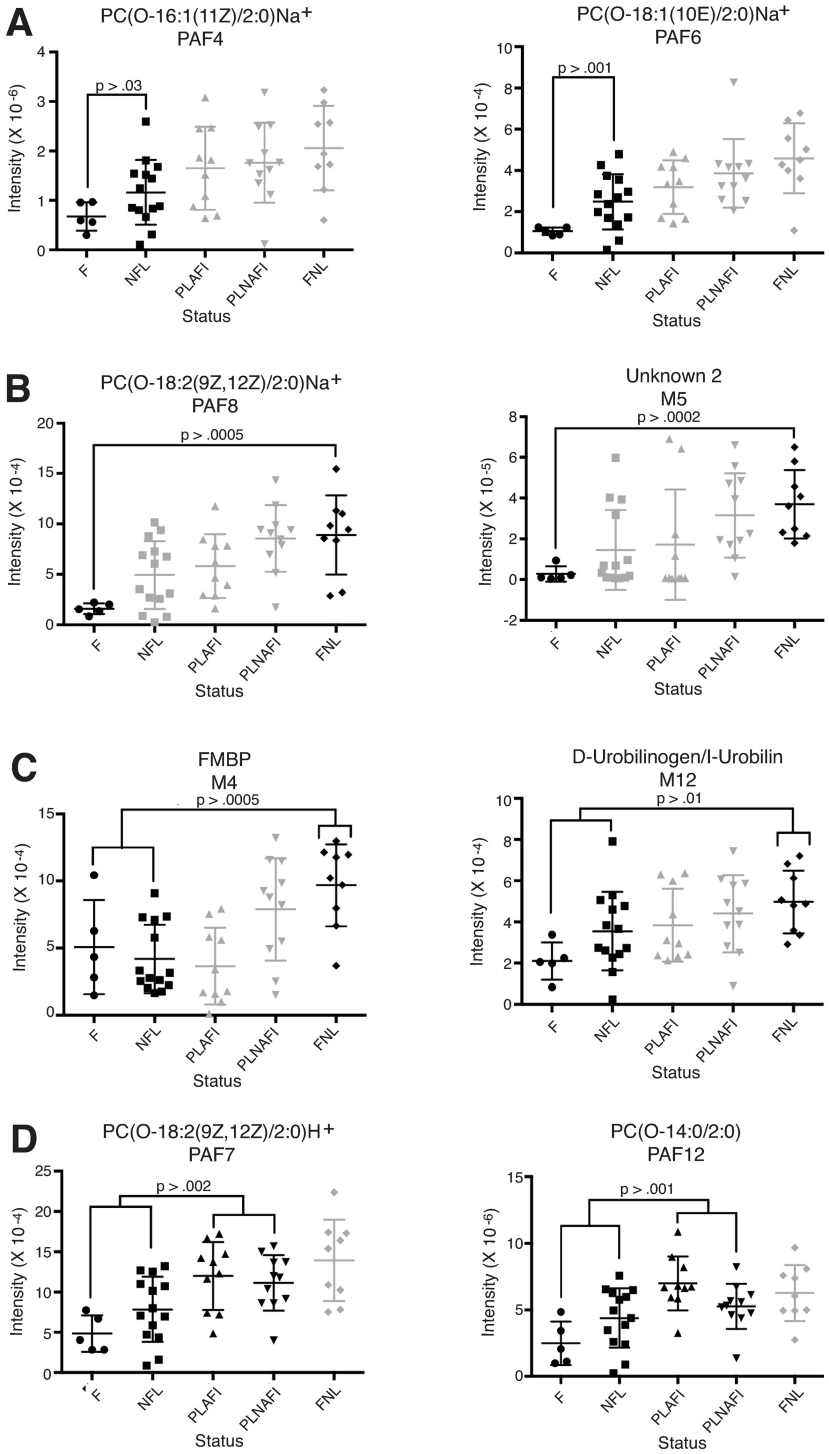
Identification of potential biomarkers for Lassa fever using machine learning. Spectral data for the biomarker candidates were extracted and analyzed by the random forests machine-learning algorithm. Examples are shown of features with a significant binary outcomes. Panel A: fatal Lassa fever versus non-Lassa febrile illnesses. Panel B: fatal versus non-Lassa febrile illness. Panel C: acute Lassa fever (fatal plus non-fatal) versus non-Lassa febrile illnesses. Panel D: acute Lassa fever (fatal plus non-fatal) versus post Lassa febrile illnesses (Acute plus non-acute). F: Fatal Lassa fever, NFL: Non-fatal Lassa fever, PLAFI: Post-Lassa fever—acute febrile illness, PLNAFI: Post-Lassa fever—non-acute febrile illness and FNL: Febrile non-Lassa illness.

Random Forest machine learning provided a quantitative assessment of the ability for metabolomics data to discriminate between patients in different serological groups. Several metabolites showed significantly different levels in different groups of patients (Fig 5). For example, PAF4 (PC(O-16:1(11Z)/2:0) H^+^, *m/z* 522.3504) and PAF6 (PC(O-18:1(10E)/2:0) H^+^, *m/z* 550.3808) were found in significantly lower levels in patients that succumbed to Lassa fever compared to those that survived acute infection (Fig 5A). PAF8 (PC(O-18:2(9Z,12Z)/2:0) H^+^, *m/z* 548.3552) and M5 (Unknown 2, *m/z* 187.0693) were found in significantly higher levels in non-Lassa febrile illness patient than in patients that succumbed to Lassa fever (Fig 5B). M4 (Fibrin monomer breakdown product Na^+^, *m/z* 168.075) and M12 (D-Urobilinogen/I-Urobilin Na^+^, *m/z* 613.3223) among other metabolites was higher in non-Lassa febrile illness patients compared to the combined groups of patients that presented with acute Lassa fever (Fatal plus nonfatal, Fig 5C). Other PAFs or PAF-like molecules, including PAF7 (PC(O-18:2(9Z,12Z)/2:0) Na^+^, *m/z* 570.3463) and PAF12 (PC(O-14:0/2:0) Na^+^, *m/z* 613.3223) distinguished acute Lassa fever patients (fatal plus nonfatal) from patients with prior LASV infection (Acute and non-acute presentations, Fig 5D).

The random forests machine-learning algorithm is also able to rank the power of the input variables in predicting sensitivity and specificity for placement in a given serological group. For example, M9 (Mesobilirubinogen H^+^, 593.3334) provided a sensitivity of 1 and a specificity of .89 when comparing fatal Lassa fever and nonfatal Lassa fever (Table 1). PAF6 (PC(O-18:1(10E)/2:0) H^+^, 550.38080) provided a sensitivity of 1 and a specificity of .78 when comparing the same groups. Receiver operator characteristic (ROC) curves for both comparisons had a value of 1 (S1A Fig). Among several metabolites with promising diagnostic potential four, PAF4 (PC(O-16:1(11Z)/2:0) H^+^, 522.3504), PAF8 (PC(O-18:2(9Z,12Z)/2:0) H^+^, 548.3552), M2 (Unknown 1 H^+^, 102.0537) and M5 (Unknown 2, 187.0693), showed sensitivities, specificities and ROC of 1 when comparing patients with a fatal outcome from Lassa fever versus nonLassa febrile illness (Table 2, S1B Fig). The algorithm was also able to identify a number of metabolites with possible diagnostic potential for discriminating between acute Lassa fever patients (fatal plus nonfatal) and non-Lassa febrile illness patients (S6 Table, S1C Fig) or patients with prior LASV infection (acute or non-acute presentation, S7 Table, S1D Fig).

**Table 1:** Diagnostic sensitivity and specificity of top biomarkers comparing fatal Lassa fever versus nonfatal Lassa fever patients.

Identifier	Descriptor	ROC	Sensitivity	Specificity
M9	Mesobilirubinogen H^+^	1	1	0.89
PAF6	PC(O-18:1(10E)/2:0) Na^+^	1	1	0.78
PAF4	PC(O-16:1(11Z)/2:0) Na^+^	0.67	1	0.78
PAF5	PC(O-18:1(10E)/2:0) H^+^	0.94	0.75	0.89
PAF8	PC(O-18:2(9Z,12Z)/2:0) Na^+^	0.86	0.75	0.89
PAF24	Lyso-PAF C-18 Na^+^	0.86	0.75	0.89
PAF23	Lyso-PAF C-18 H^+^	0.75	0.75	0.89
M12	D-Urobilinogen/I-Urobilin Na^+^	0.79	0.5	0.83
PAF22	PAF C-18 Na^+^	0.56	0.5	0.83
PAF3	PC(O-16:1(11Z)/2:0) H^+^	1	0.5	0.78
PAF12	PC(O-14:0/2:0) Na^+^	0.945	0.5	0.78
PAF20	Arachidonoyl PAF C-16 Na^+^	0.86	0.5	0.78
PAF7	PC(O-18:2(9Z,12Z)/2:0) H^+^	0.71	0.5	0.72
PAF19	Arachidonoyl PAF C-16 H^+^	0.42	0.5	0.72
M18	1-Methylinosine Na^+^	1	0.5	0.67
PAF16	PAF C-16 Na^+^	0.635	0.5	0.67
M10	Mesobilirubinogen Na^+^	0.57	0.5	0.67
M11	D-Urobilinogen/I-Urobilin H^+^	0.71	0.5	0.56
PAF11	PC(O-14:0/2:0) H^+^	0.33	0.5	0.56
PAF17	Lyso-PAF C-16 H^+^	0.79	0.38	0.67
PAF14	PC(O-15:0/2:0) Na^+^	0.5	0.38	0.61
PAF10	PC(O-12:0/2:0) Na^+^	0.75	0.25	0.78
PAF9	PC(O-12:0/2:0) H^+^	0.71	0.25	0.72
M17	1-Methylinosine H^+^	0.64	0.25	0.67
PAF15	PAF C-16 H^+^	0.5	0.25	0.44
PAF13	PC(O-15:0/2:0) H^+^	0.25	0.25	0.44
M5	Unknown 2	0.36	0.13	0.67
PAF21	PAF C-18 H^+^	0.63	0.13	0.56
PAF18	Lyso-PAF C-16 Na^+^	0.88	0	0.78
M4	FMBP	0.063	0	0.78
PAF2	PC(O-10:1(9E)/2:0) Na^+^	0.25	0	0.5
PAF1	PC(O-10:1(9E)/2:0) H^+^	0.25	0	0.44

**Table 2:** Diagnostic sensitivity and specificity of top biomarkers comparing fatal Lassa fever versus non Lassa febrile illness patients.

Identifier	Descriptor	ROC	Sensitivity	Specificity
PAF4	PC(O-16:1(11Z)/2:0) Na^+^	1	1	1
PAF8	PC(O-18:2(9Z,12Z)/2:0) Na^+^	1	1	1
M2	Unknown 1 H^+^	1	1	1
M5	Unknown 2	1	1	1
PAF3	PC(O-16:1(11Z)/2:0) H^+^	1	1	0.86
PAF21	PAF C-18 H^+^	0.93	1	0.86
M9	Mesobilirubinogen H^+^	0.8	1	0.86
PAF7	PC(O-18:2(9Z,12Z)/2:0) H^+^	1	0.75	1
PAF12	PC(O-14:0/2:0) Na^+^	1	0.75	1
M10	Mesobilirubinogen Na^+^	1	0.75	1
PAF10	PC(O-12:0/2:0) Na^+^	1	0.75	0.86
M18	1-Methylinosine Na^+^	1	0.75	0.86
PAF5	PC(O-18:1(10E)/2:0) H^+^	0.93	0.75	0.86
PAF24	Lyso-PAF C-18 Na^+^	0.92	0.75	0.86
PAF17	Lyso-PAF C-16 H^+^	1	0.75	0.71
PAF18	Lyso-PAF C-16 Na^+^	1	0.75	0.71
M12	D-Urobilinogen/I-Urobilin Na^+^	1	0.75	0.71
M11	D-Urobilinogen/I-Urobilin H^+^	0.9	0.63	0.86
PAF6	PC(O-18:1(10E)/2:0) Na^+^	0.88	0.63	0.86
PAF9	PC(O-12:0/2:0) H^+^	0.86	0.63	0.71
PAF2	PC(O-10:1(9E)/2:0) Na^+^	1	0.5	0.86
PAF23	Lyso-PAF C-18 H^+^	0.92	0.5	0.86
PAF16	PAF C-16 Na^+^	0.7	0.5	0.79
PAF13	PC(O-15:0/2:0) H^+^	0.5	0.5	0.78
PAF22	PAF C-18 Na^+^	0.9	0.5	0.71
M17	1-Methylinosine H^+^	0.9	0.5	0.71
PAF1	PC(O-10:1(9E)/2:0) H^+^	0.83	0.5	0.71
PAF20	Arachidonoyl PAF C-16 Na^+^	0.8	0.5	0.71
M3	Unknown 1 NH_4_^+^	0.71	0.5	0.71
PAF14	PC(O-15:0/2:0) Na^+^	0.38	0.5	0.71
M4	FMBP	0.7	0.38	0.71
PAF15	PAF C-16 H^+^	0.83	0.25	0.71
PAF19	Arachidonoyl PAF C-16 H^+^	0.42	0.25	0.5
PAF11	PC(O-14:0/2:0) H^+^	0.42	0	0.64

## Discussion

LASV induces a dynamic physiological dysregulation within the circulatory system of infected humans, which is manifest in changes in the levels of numerous metabolites. Pathways mediating blood coagulation, hemoglobin breakdown and lipid, amino acid, nucleic acid metabolism are affected during or following LASV infection. Further investigation of these metabolic pathways may inform discovery of novel therapeutic targets for Lassa fever. Metabolites that differentiate Lassa fever patients at various stages of disease, as well those that differentiated these patients from other febrile illness patients presenting to KGH, have been identified. Several compounds, including PAF, PAF-like molecules and products of heme breakdown emerged as candidates that may prove useful in diagnostic assays to inform better care of Lassa fever patients.

Several PAFs or PAF-like molecules demonstrated high sensitivity and specificity for discriminating between patients that ultimately succumbed to fatal Lassa fever and those that survived. The primary physiological role of platelets is to aggregate at the site of endothelial injury where they initiate the clotting cascade to block circulatory leak [48]. Human and nonhuman primates infected with LASV develop clotting abnormalities that manifest in abnormal *in vitro* platelet aggregation [29,39,49,50]. Levels of platelets in the blood and platelet survival times are normal or only slightly depressed in Lassa patients. Abnormal platelet aggregation correlated with the presence of hemorrhage and with the severity of disease. Here, we demonstrate that levels of PAF or PAF-like molecules were decreased in Lassa fever patients that succumbed to their infection. An as yet to be identified inhibitor of platelet aggregation was identified in the blood of patients with Lassa fever as well as the in patients with Argentine hemorrhagic fever, which is caused by Junin virus, an arenavirus related to LASV [47,51]. The contribution of PAF and PAF-like molecules to hemorrhagic fever pathogenesis appears to be complex. In dengue virus infected patients, PAF appears to be a contributing factor to vascular leakage [52] and higher expression of PAF-degrading acetylhydrolase (PAF-AH) correlates with lower frequency of dengue fever, but not dengue hemorrhagic fever in two ethnically distinct populations [53]. In a murine model of dengue genetic knockout or chemical inhibition of the platelet-activating factor receptor (PAFR) resulted in a less severe disease and increased survival in those animals deficient or inhibited for PAFR [54]. Additional studies will be required to determine if decreased PAF mediated platelet activation contributes to the hemorrhagic manifestations of severe Lassa fever, and whether or PAFs or PAF-like molecules can serve as diagnostic or prognostic markers.

Hemoglobin breakdown products were identified as potential prognostic biomarkers. Mesobilirubinogen (M9) exhibited a specificity of 1 and sensitivity of >86% in discriminating between fatal Lassa fever and either non-fatal Lassa fever or non-Lassa febrile illness. Two confounding factors concerning the presence of heme breakdown products are worth noting for consideration in future studies. First is the malaria endemic locale where over 75% of febrile patients test positive for *Plasmodium spp* [25]. Second, 35% of the LF patients received ribavirin treatment, a drug attributed to development of anemia [55].

In the present study, serum lipids were the most frequently identified molecular class and also the most frequently identified as decreased in fatal Lassa fever. Similar results were obtained previously in a study of lymphocytic choriomeningitis virus (LCMV) infected mice, a small animal model of arenavirus infection. Stearoyl lysophosphocholine (18:0), identified with positive ion *m/z* 524.37057, had reduced signal intensity in the serum of infected animals [43]. Proteolytic breakdown products are also observed in this murine arenavirus infection model. The dipeptides γ-glutamyl-Valine, γ-glutamyl-Leucine, and prolyl-hydroxyproline were present in lower amounts in the plasma over the course of LCMV infection in mice [43]. We observed both increased and decreased amounts of a several peptide species. However, no consistent pattern emerged to suggest a mechanism that might account for differences in Lassa fever patients, survivors or febrile controls. Despite the observed differences amongst patient groups there were no peptide or lipid species identified that showed diagnostic sensitivity and specificity approaching that of PAFs or heme breakdown products. In this regard, additional features in the present dataset merit continued investigation to obtain a definite chemical identification. Unknown 1 (H^+^ *m/z* 102.0537, NH_4_^+^ *m/z* 119.08) was significantly elevated in serum samples from patients with fatal Lassa fever compared to those with non-Lassa febrile illness. A second unknown spectral feature detected at *m/z* 187.0693 showed a significant reduction in sera from Lassa fever patients that died compared to non-Lassa febrile patients.

Virus load, the levels of liver enzymes and certain cytokines have predictive value in the outcomes of Lassa fever [28,35,56,57]. Assays to measure these parameters are generally not feasible in austere environments. Many rural health posts across the Lassa fever zone in West Africa are challenged by lack of electricity, minimal lab infrastructure and lack of access to training. LCMS technology is also not feasible in field clinics in West Africa where Lassa fever patients are prevalent. Therefore, simple assays such as dipstick style chromatographic assays, including lateral flow immunoassays, have gained acceptance, and can be conducted with minimal resources and training [35,58–60]. Several small molecules with high biomarker potential were identified including adducts of the modified nucleoside 1-methylinosine. The protonated, sodiated, and potassiated adducts of 1-methylinosine are elevated in the urine of cancer patients [61,62] and in the plasma of patients in renal failure whereupon removal possesses biomarker utility for effective hemodialysis [63]. Reagents used in urinalysis sticks for assaying 1-methylinosine, and similar approaches for quantifying heme breakdown products and other metabolites potentially could be adapted for a Lassa fever prognostic/diagnostic panel.

While these studies have putatively identified a number of compounds with altered serum levels during LASV disease, unequivocal identity of a compound by LCMS requires comparison of the spectrum of the metabolite with a reference standard, which will be pursued for molecules with diagnostic potential. Furthermore, the panel of candidate biomarkers, including PAF, PAF-like molecules and heme breakdown products, must be investigated for diagnostic efficacy alone and in combination in prospective clinical studies. Another limitation of the current study is the relatively low numbers of patients samples analyzed. Additional metabolomics profiling using a larger number of patient samples should be conducted, including LCMS analysis of metabolites in noninvasive specimens such as saliva or urine. Larger panels including other bodily fluids may expand the panel of potential metabolites with diagnostic potential. Metabolic markers for early diagnosis and prognosis of patients at high risk for development of fatal Lassa fever could be integrated into existing clinical and laboratory algorithms for LASV diagnosis and prognosis and improve outcomes of this often fatal disease by identifying cases at greatest risk of death. In addition, the application of metabolomics to reveal fundamental LASV pathogenic mechanisms will potentially provide new targets for therapeutic interventions.

## Supporting information

S1 Table Demographics of patients presenting to the Kenema Government Hospital Viral Hemorrhagic Fever Ward. (DOCX)

S2 Table Platelet-activating factor (PAF) and PAF-like serum metabolites detected in serum of febrile patients presenting to the Kenema Government Hospital Viral Hemorrhagic Fever Ward. (DOCX)

S3 Table Miscellaneous serum metabolites detected in serum of febrile patients presenting to the Kenema Government Hospital Viral Hemorrhagic Fever Ward. (DOCX)

S4 Table Peptides detected in serum of febrile patients presenting to the Kenema Government Hospital Viral Hemorrhagic Fever Ward. (DOCX)

S5 Table Lipids detected in serum of febrile patients presenting to the Kenema Government Hospital Viral Hemorrhagic Fever Ward.^1^ (DOCX)

S6 Table Diagnostic sensitivity and specificity of top biomarkers comparing Lassa fever (fatal plus nonfatal) versus non Lassa febrile illness patients. (DOCX)

S7 Table Diagnostic sensitivity and specificity of top biomarkers comparing Lassa fever (fatal plus nonfatal) versus post Lassa (acute plus nonacute) patients. (DOCX)

S1 Fig Receiver operator characteristic curves for indicated metabolites. Panel A: fatal Lassa fever versus non-Lassa febrile illnesses. Panel B: fatal versus non-Lassa febrile illness. Panel C: acute Lassa fever (fatal plus non-fatal) versus non-Lassa febrile illnesses. Panel D: acute Lassa fever (fatal plus non-fatal) versus post Lassa febrile illnesses (Acute plus non-acute).
(TIFF)
