# Supplementary material for: Metabolomics analyses identify platelet activating factors and heme breakdown products as Lassa fever biomarkers
Source: PLoS Negl Trop Dis. 2017 Sep 18;11(9):e0005943. doi: 10.1371/journal.pntd.0005943 (PMC5619842; doi:10.1371/journal.pntd.0005943)
Supplement: S1 Table — (DOCX) [file pntd.0005943.s001.docx]

S1 Table. Demographics of patients presenting to the Kenema Government Hospital Lassa Fever Ward.

| G number | Diagnosis | Sex | Age | Outcome |
| --- | --- | --- | --- | --- |
| G-2349-1 | Fatal Lassa fever^1^ | F | 20 | Died |
| G-2557-1 | Fatal Lassa fever | F | 38 | Died |
| G-2612-1 | Fatal Lassa fever | M | 24 | Died |
| G-2615-1 | Fatal Lassa fever | M | 32 | Died |
| G-2770-1 | Fatal Lassa fever | - | - | Died |
| G-2319-1 | Non Fatal Lassa fever^1^ | F | 24 | Discharged |
| G-2325-1 | Non Fatal Lassa fever | F | 4 | Not admitted |
| G-2380-1 | Non Fatal Lassa fever | F | 43 | Not admitted |
| G-2387-1 | Non Fatal Lassa fever | M | 23 | Discharged |
| G-2424-1 | Non Fatal Lassa fever | F | 7 | Discharged |
| G-2423-1 | Non Fatal Lassa fever | M | 9 | Discharged |
| G-2511-1 | Non Fatal Lassa fever | F | 22 | Discharged |
| G-2554-1 | Non Fatal Lassa fever | F | 39 | Discharged |
| G-2560-1 | Non Fatal Lassa fever | M | 35 | Not admitted |
| G-2587-1 | Non Fatal Lassa fever | M | 6 | Discharged |
| G-2585-1 | Non Fatal Lassa fever | F | 38 | Not admitted |
| G-2614-1 | Non Fatal Lassa fever | F | 43 | Discharged |
| G-2727-1 | Non Fatal Lassa fever | F | 40 | Not admitted |
| G-2740-1 | Non Fatal Lassa fever | F | 20 | Discharged^2^ |
| G-2429-1 | Post-Lassa Acute Febrile Illness^3^ | M | 20 | Not admitted |
| G-2432-1 | Post-Lassa Acute Febrile Illness | F | 21 | Not admitted |
| G-2444-1 | Post-Lassa Acute Febrile Illness | M | 20 | Discharged |
| G-2446-1 | Post-Lassa Acute Febrile Illness | M | 65 | Not admitted |
| G-2541-1 | Post-Lassa Acute Febrile Illness | M | 38 | Not admitted |
| G-2607-1 | Post-Lassa Acute Febrile Illness | M | 10 | Not admitted |
| G-2619-1 | Post-Lassa Acute Febrile Illness | M | 20 | Not admitted |
| G-2617-1 | Post-Lassa Acute Febrile Illness | F | 28 | Not admitted |
| G-2755-1 | Post-Lassa Acute Febrile Illness | - | - | Not admitted |
| G-2733-1 | Post-Lassa Acute Febrile Illness | F | 19 | Discharged |
| G-2326-1 | Post-Lassa Non Acute Febrile Illness^3^ | F | 21 | Not admitted |
| G-2433-1 | Post-Lassa Non Acute Febrile Illness | M | 32 | Not admitted |
| G-2437-1 | Post-Lassa Non Acute Febrile Illness | F | 18 | Not admitted |
| G-2466-1 | Post-Lassa Non Acute Febrile Illness | M | 56 | Discharged |
| G-2552-1 | Post-Lassa Non Acute Febrile Illness | F | 21 | Discharged |
| G-2559-1 | Post-Lassa Non Acute Febrile Illness | F | 20 | Not admitted |
| G-2622-1 | Post-Lassa Non Acute Febrile Illness | M | 42 | Not admitted |
| G-2660-1 | Post-Lassa Non Acute Febrile Illness | - | - | Not admitted |
| G-2724-1 | Post-Lassa Non Acute Febrile Illness | F | 25 | Not admitted |
| G-2731-1 | Post-Lassa Non Acute Febrile Illness | F | 19 | Discharged |
| G-2725-1 | Post-Lassa Non Acute Febrile Illness | M | 33 | Not admitted |
| G-2299-1 | Non Lassa fever^4^ | F | 9 | Died |
| G-2308-1 | Non Lassa fever | M | 30 | Not admitted |
| G-2314-1 | Non Lassa fever | F | 11 | Not admitted |
| G-2370-1 | Non Lassa fever | F | 19 | Died |
| G-2402-1 | Non Lassa fever | F | 40 | Not admitted |
| G-2449-1 | Non Lassa fever | M | 40 | Not admitted |
| G-2561-1 | Non Lassa fever | F | 18 | Not admitted |
| G-2726-1 | Non Lassa fever |  |  | Not admitted |
| G-2746-1 | Non Lassa fever |  |  | Not admitted |

| ^1^Ag+ or PCR+ |
| --- |
| ^2^Discharged against medical advice |
| ^3^Ag-, PCR-, IgM or IgG+ |
| ^4^Ag-, PCR-, IgM-, and IgG- |
