# Supplementary material for: Metabolomics analyses identify platelet activating factors and heme breakdown products as Lassa fever biomarkers
Source: PLoS Negl Trop Dis. 2017 Sep 18;11(9):e0005943. doi: 10.1371/journal.pntd.0005943 (PMC5619842; doi:10.1371/journal.pntd.0005943)
Supplement: S2 Table — (DOCX) [file pntd.0005943.s002.docx]

S2 Table. Platelet-activating factor (PAF) and PAF-like serum metabolites detected in serum of febrile patients presenting to the Kenema Government Hospital Viral Hemorrhagic Fever Ward.^1^

| Identifier | Descriptor | Observed *m/z* |  |
| --- | --- | --- | --- |
| PAF11 | PC(O-14:0/2:0) H^+^ | 496.3352 |  |
| PAF12 | PC(O-14:0/2:0) Na^+^ | 518.3167 |  |
| PAF15 | PAF C-16 H^+^ | 524.3659 |  |
| PAF16 | PAF C-16 Na^+^ | 546.3475 |  |
| PAF4 | PC(O-16:1(11Z)/2:0) Na^+^ | 544.3327 |  |
| PAF3 | PC(O-16:1(11Z)/2:0) H^+^ | 522.3504 |  |
| PAF18 | Lyso-PAF C-16 Na^+^ | 504.3373 |  |
| PAF7 | PC(O-18:2(9Z,12Z)/2:0) H^+^ | 548.3552 |  |
| PAF20 | Arachidonoyl PAF C-16 Na^+^ | 790.5632 |  |
| PAF17 | Lyso-PAF C-16 H^+^ | 482.3557 |  |
| PAF13 | PC(O-15:0/2:0) H^+^ | 510.35 |  |
| PAF14 | PC(O-15:0/2:0) Na^+^ | 532.3317 |  |
| PAF24 | Lyso-PAF C-18 Na^+^ | 532.3672 |  |
| PAF6 | PC(O-18:1(10E)/2:0) Na^+^ | 572.3629 |  |
| PAF9 | PC(O-12:0/2:0) H^+^ | 468.3034 |  |
| PAF1 | PC(O-10:1(9E)/2:0) H^+^ | 438.2934 |  |
| PAF5 | PC(O-18:1(10E)/2:0) H^+^ | 550.3808 |  |
| PAF10 | PC(O-12:0/2:0) Na^+^ | 490.2852 |  |
| PAF22 | PAF C-18 Na^+^ | 552.3945 |  |
| PAF21 | PAF C-18 H^+^ | 574.3759 |  |
| PAF2 | PC(O-10:1(9E)/2:0) Na^+^ | 460.2754 |  |
| PAF19 | Arachidonoyl PAF C-16 H^+^ | 768.579 |  |
| PAF23 | Lyso-PAF C-18 H^+^ | 510.3858 |  |
| PAF8 | PC(O-18:2(9Z,12Z)/2:0) Na^+^ | 570.3463 |  |

^1^Metabolites are listed in the order (top to bottom) of appearance in Fig. 2A.
