# Supplementary material for: Metabolomics analyses identify platelet activating factors and heme breakdown products as Lassa fever biomarkers
Source: PLoS Negl Trop Dis. 2017 Sep 18;11(9):e0005943. doi: 10.1371/journal.pntd.0005943 (PMC5619842; doi:10.1371/journal.pntd.0005943)
Supplement: S3 Table — (DOCX) [file pntd.0005943.s003.docx]

S3 Table. Miscellaneous serum metabolites detected in serum of febrile patients presenting to the Kenema Government Hospital Viral Hemorrhagic Fever Ward.^1^

| Identifier | Descriptor | Observed *m/z* |  |
| --- | --- | --- | --- |
| M4 | Fibrin monomer breakdown product Na^+^ | 168.075 |  |
| M10 | Mesobilirubinogen Na^+^ | 615.3373 |  |
| M11 | D-Urobilinogen/I-Urobilin H^+^ | 591.3195 |  |
| M12 | D-Urobilinogen/I-Urobilin Na^+^ | 613.3223 |  |
| M15 | Caffeoylputrescine H^+^ | 251.1341 |  |
| M3 | Unknown 1 NH_4_^+^ | 119.08 |  |
| M8 | D-Dopachrome Na^+^ | 216.0278 |  |
| M14 | Methyluridine H^+^ | 259.0906 |  |
| M9 | Mesobilirubinogen H^+^ | 593.3334 |  |
| M1 | Phosphohydroxypyruvic acid H^+^ | 184.9834 |  |
| M13 | L-Urobilin H^+^ | 595.3493 |  |
| M2 | Unknown 1 H^+^ | 102.0537 |  |
| M17 | 1-Methylinosine H^+^ | 283.1037 |  |
| M16 | Caffeoylputrescine Na^+^ | 273.1185 |  |
| M6 | N-formylanthranilic acid Na^+^ | 188.0331 |  |
| M7 | D-Dopachrome H^+^ | 194.0463 |  |
| M18 | 1-Methylinosine Na^+^ | 305.0856 |  |
| M5 | Unknown 2 | 187.0693 |  |

^1^Metabolites are listed in the order (top to bottom) of appearance in Fig. 2B.
