# Supplementary material for: Metabolomics analyses identify platelet activating factors and heme breakdown products as Lassa fever biomarkers
Source: PLoS Negl Trop Dis. 2017 Sep 18;11(9):e0005943. doi: 10.1371/journal.pntd.0005943 (PMC5619842; doi:10.1371/journal.pntd.0005943)
Supplement: S4 Table — (DOCX) [file pntd.0005943.s004.docx]

S4 Table. Peptides detected in serum of febrile patients presenting to the Kenema Government Hospital Viral Hemorrhagic Fever Ward.^1^

| Identifier | Descriptor | Observed *m/z* |
| --- | --- | --- |
| P34 | Pro Trp Gly K^+^ | 397.1262 |
| P26 | Glu Glu Cys H^+^ | 380.1144 |
| P33 | Glutamylalanine Na^+^ | 241.0799 |
| P21 | Arg Trp Arg H^+^ | 517.3036 |
| P8 | Phe Arg Arg Na^+^ | 500.2696 |
| P3 | Phe Arg Arg H^+^ | 478.2876 |
| P28 | Trp Lys Ala Na^+^ | 426.2120 |
| P32 | Gln Gly Asp Na^+^ | 341.1043 |
| P14 | Cys Trp Ala K^+^ | 417.0965 |
| P10 | Asp Pro Asp H^+^ | 346.1217 |
| P6 | Gln Thr Ala H^+^ | 319.1582 |
| P27 | Phe Phe Ile Na^+^ | 448.2241 |
| P22 | Alanyl-Glutamine Na^+^ | 240.0957 |
| P31 | Prolylhydroxyproline H^+^ | 229.1164 |
| P13 | γ-Glutamyl-β-aminopropiononitrile H^+^ | 200.1012 |
| P11 | Phe Trp Phe K^+^ | 537.1860 |
| P29 | Arg Arg Thr Na^+^ | 454.2464 |
| P7 | L-Threonine K^+^ | 158.0226 |
| P9 | Lys Trp Cys H^+^ | 436.1992 |
| P19 | Met Gln Asn Na^+^ | 414.1408 |
| P24 | Asn Cys Ala H^+^ | 307.1072 |
| P25 | Asn Cys Ala Na^+^ | 329.0887 |
| P23 | Isoleucyl-Methionine Na^+^ | 285.1249 |
| P12 | Trp-Asp-OH Na^+^ | 464.1049 |
| P2 | L-alpha-Aspartyl-L-hydroxyproline H^+^ | 247.0908 |
| P15 | Lys-TyrMe-OH K^+^ | 470.1285 |
| P18 | Phe-Trp-OH Na^+^ | 482.1306 |
| P17 | TyrMe-Phe-OH H^+^ | 465.1641 |
| P5 | Cys Asn His Na^+^ | 395.1140 |
| P4 | Gly Met Lys K^+^ | 373.1323 |
| P20 | Val Arg Arg H^+^ | 430.2907 |
| P1 | L-beta-Aspartyl-L-threonine Na^+^ | 257.0722 |
| P16 | Methionyl-Valine K^+^ | 287.0830 |
| P30 | Arginyl-Proline Na^+^ | 294.1523 |

^1^Peptides are listed in the order (top to bottom) of appearance in Fig. 2C.
