# Supplementary material for: Metabolomics analyses identify platelet activating factors and heme breakdown products as Lassa fever biomarkers
Source: PLoS Negl Trop Dis. 2017 Sep 18;11(9):e0005943. doi: 10.1371/journal.pntd.0005943 (PMC5619842; doi:10.1371/journal.pntd.0005943)
Supplement: S5 Table — (DOCX) [file pntd.0005943.s005.docx]

S5 Table. Lipids detected in serum of febrile patients presenting to the Kenema Government Hospital Viral Hemorrhagic Fever Ward.^1^

| Identifier | Descriptor | Observed *m/z* |  |
| --- | --- | --- | --- |
| L118 | Taurocholic acid H^+^ | 516.3012 |  |
| L90 | PA(12:0/15:1(9Z)) K^+^ | 615.3373 |  |
| L27 | PI(O-20:0/14:1(9Z)) K^+^ | 861.5221 |  |
| L1 | all-trans-nonaprenyl diphosphate H^+^ | 791.5195 |  |
| L77 | 13-sophorosyloxydocosanoic acid Na^+^ | 703.4191 |  |
| L37 | DG(17:1(9Z)/22:3(10Z,13Z,16Z)/0:0)[iso2] K^+^ | 697.5175 |  |
| L23 | PE(18:1(11Z)/P-18:1(11Z)) H^+^ | 728.5632 |  |
| L78 | PE(16:0/0:0) Na^+^ | 476.2702 |  |
| L138 | 30:4(15Z,18Z,21Z,24Z) K^+^ | 483.3588 |  |
| L146 | PS(16:0/20:0) H^+^ | 792.5699 |  |
| L51 | PC(O-18:0/22:6(4Z,7Z,10Z,13Z,16Z,19Z)) K^+^ | 858.5826 |  |
| L99 | NeuAcalpha2-3Galbeta-Cer(d18:1/16:0) H^+^ | 991.6605 |  |
| L2 | PA(P-20:0/21:0) Na^+^ | 781.6062 |  |
| L31 | PC(22:6(4Z,7Z,10Z,13Z,16Z,19Z)/22:6(4Z,7Z,10Z,13Z,16Z,19Z)) H^+^ | 878.5743 |  |
| L12 | PA(P-20:0/19:1(9Z)) K^+^ | 767.5380 |  |
| L136 | PE(O-18:0/16:0)[U] K^+^ | 744.5303 |  |
| L20 | PA(O-16:0/19:1(9Z)) K^+^ | 713.4918 |  |
| L115 | PS(18:1(9Z)/18:1(9Z))[U] H^+^ | 788.5477 |  |
| L97 | stigmast-5-en-3beta-ol 3-0-beta-D-glucopyranoside Na^+^ | 599.4331 |  |
| L69 | N,N,N-trimethyl-sphingosine H^+^ | 342.3334 |  |
| L98 | 6α-Glucuronosylhyodeoxycholate H^+^ | 569.3362 |  |
| L119 | PC(16:1(9E)/0:0) H^+^ | 494.3192 |  |
| L67 | PC(22:6(4E,7E,10E,13E,16E,19E)/0:0)[U] Na^+^ | 590.3165 |  |
| L42 | PC(22:6(4Z,7Z,10Z,13Z,16Z,19Z)/22:6(4Z,7Z,10Z,13Z,16Z,19Z)) Na^+^ | 900.5592 |  |
| L55 | DG(21:0/22:5(7Z,10Z,13Z,16Z,19Z)/0:0)[iso2] K^+^ | 751.5641 |  |
| L56 | PIP(16:0/22:5(4Z,7Z,10Z,13Z,16Z)) H^+^ | 965.5188 |  |
| L101 | PS(16:0/18:1(11Z)) K^+^ | 800.4768 |  |
| L74 | C25:4 Highly branched isoprenoid A Na^+^ | 367.3362 |  |
| L95 | 1α-hydroxy-25-methoxyvitamin D3 / 1α-hydroxy-25-methoxycholecalciferol K^+^ | 469.3071 |  |
| L94 | PI-Cer(t18:0/18:0(2OH)) K^+^ | 880.5384 |  |
| L109 | PE(18:2(9Z,12Z)/18:2(9Z,12Z))[U] K^+^ | 778.4810 |  |
| L117 | PA(O-20:0/0:0) K^+^ | 491.2886 |  |
| L116 | PC(17:1(10Z)/0:0) Na^+^ | 530.3173 |  |
| L40 | 2,4,6-trimethyl-2,15-tetracosadienoic acid Na^+^ | 429.3704 |  |
| L61 | Ubiquinone 8 Na^+^ | 749.5486 |  |
| L36 | PG(20:0/22:1(11Z)) K^+^ | 899.6100 |  |
| L14 | PI(O-16:0/18:2(9Z,12Z)) K^+^ | 859.5064 |  |
| L30 | PS(P-16:0/20:5(5Z,8Z,11Z,14Z,17Z)) H^+^ | 766.5066 |  |
| L13 | PG(19:0/21:0) K^+^ | 873.5947 |  |
| L142 | 1α-fluoro-25-hydroxy-16,17,23,23,24,24-hexadehydrovitamin D3 H^+^ | 413.2839 |  |
| L84 | PA(O-18:0/17:0) Na^+^ | 699.5270 |  |
| L125 | PI(22:1(11Z)/22:2(13Z,16Z)) K^+^ | 1,011.6281 |  |
| L106 | PG(15:1(9Z)/22:6(4Z,7Z,10Z,13Z,16Z,19Z)) H^+^ | 779.4895 |  |
| L153 | PS(15:1(9Z)/22:4(7Z,10Z,13Z,16Z)) K^+^ | 834.4693 |  |
| L123 | PG(14:1(9Z)/14:1(9Z)) H^+^ | 663.4250 |  |
| L105 | PS(13:0/22:2(13Z,16Z)) K^+^ | 812.4766 |  |
| L110 | PS(17:0/22:2(13Z,16Z)) K^+^ | 868.5503 |  |
| L38 | PI(18:3(6Z,9Z,12Z)/22:6(4Z,7Z,10Z,13Z,16Z,19Z)) Na^+^ | 927.4937 |  |
| L83 | PA(O-20:0/20:5(5Z,8Z,11Z,14Z,17Z)) H^+^ | 737.5493 |  |
| L68 | Nonadecyl oleate Na^+^ | 543.5112 |  |
| L103 | PG(18:3(6Z,9Z,12Z)/20:5(5Z,8Z,11Z,14Z,17Z)) H^+^ | 791.4894 |  |
| L26 | PE(O-16:0/16:0)[U] K^+^ | 716.4965 |  |
| L28 | PA(O-16:0/17:1(9Z)) K^+^ | 685.4585 |  |
| L62 | PIP(18:2(9Z,12Z)/20:1(11Z)) H^+^ | 969.5494 |  |
| L137 | MG(0:0/20:4(5Z,8Z,11Z,14Z)/0:0) Na^+^ | 401.2630 |  |
| L129 | PE(20:5(5Z,8Z,11Z,14Z,17Z)/24:1(15Z)) K^+^ | 886.5737 |  |
| L121 | 34:5(19Z,22Z,25Z,28Z,31Z) K^+^ | 537.4042 |  |
| L120 | 36:5(21Z,24Z,27Z,30Z,33Z) K^+^ | 565.4349 |  |
| L64 | PG(17:0/19:0) K^+^ | 817.5359 |  |
| L114 | PC(15:0/20:4(5Z,8Z,11Z,14Z))[U] K^+^ | 806.5131 |  |
| L113 | PG(17:1(9Z)/22:6(4Z,7Z,10Z,13Z,16Z,19Z)) H^+^ | 807.5221 |  |
| L111 | Ganglioside GM3 (d18:1/23:0) Na^+^ | 1,273.8073 |  |
| L45 | PS(22:6(4Z,7Z,10Z,13Z,16Z,19Z)/20:4(5Z,8Z,11Z,14Z)) K^+^ | 894.4719 |  |
| L35 | PIP(16:1(9Z)/16:1(9Z)) Na^+^ | 909.4429 |  |
| L63 | PIP(16:0/22:4(10Z,13Z,16Z,19Z)) H^+^ | 967.5332 |  |
| L147 | Hexadecanedioic acid mono-L-carnitine ester H^+^ | 430.3124 |  |
| L148 | Leucettamol A K^+^ | 511.3673 |  |
| L48 | 1-Palmitoyl-2-(5-hydroxy-8-oxo-6-octenedioyl)-sn-glycero-3-phosphatidylcholine Na^+^ | 688.3858 |  |
| L49 | PI(16:0/18:2(9Z,12Z)) H^+^ | 835.5256 |  |
| L46 | PG(17:2(9Z,12Z)/22:6(4Z,7Z,10Z,13Z,16Z,19Z)) Na^+^ | 827.4867 |  |
| L152 | PA(12:0/19:0) K^+^ | 673.4170 |  |
| L135 | Anandamide (20:3, n-3) Na^+^ | 372.2867 |  |
| L151 | PS(16:0/0:0) H^+^ | 498.2829 |  |
| L102 | PE(18:0/0:0) H^+^ | 482.3200 |  |
| L104 | (6R)-vitamin D3 6,19-(4-phenyl-1,2,4-triazoline-3,5-dione) adduct K^+^ | 598.3391 |  |
| L108 | 4,4-Dimethyl-14a-hydroxymethyl-5a-cholesta-8,24-dien-3b-ol K^+^ | 481.3430 |  |
| L89 | Cholest-5-ene H^+^ | 371.3676 |  |
| L71 | 3-Deoxyvitamin D3 H^+^ | 369.3513 |  |
| L144 | PI(O-20:0/20:3(8Z,11Z,14Z)) H^+^ | 903.6379 |  |
| L16 | PI(16:0/16:2(9Z,12Z)) H^+^ | 807.4938 |  |
| L124 | Cholesta-5,7-diene-1,3-diol H^+^ | 401.3408 |  |
| L88 | Geranyl diphosphate H^+^ | 315.0771 |  |
| L21 | PC(14:0/18:3(9Z,12Z,15Z)) H^+^ | 728.5282 |  |
| L128 | PE(14:1(9Z)/18:4(6Z,9Z,12Z,15Z)) Na^+^ | 704.4231 |  |
| L79 | PS(19:1(9Z)/0:0) K^+^ | 576.2742 |  |
| L91 | PS(P-18:0/22:6(4Z,7Z,10Z,13Z,16Z,19Z)) H^+^ | 820.5523 |  |
| L85 | PS(13:0/20:3(8Z,11Z,14Z)) K^+^ | 782.4355 |  |
| L3 | 20:3-Glc-cholesterol K^+^ | 875.6103 |  |
| L131 | PI(14:1(9Z)/14:1(9Z)) Na^+^ | 773.4206 |  |
| L122 | 32:4(17Z,20Z,23Z,26Z) K^+^ | 511.3894 |  |
| L132 | PI-Cer(d18:0/20:0) Na^+^ | 860.5987 |  |
| L96 | PA(17:0/20:4(5Z,8Z,11Z,14Z)) K^+^ | 766.4836 |  |
| L107 | NeuAcalpha2-3Galbeta-Cer(d18:1/20:0) Na^+^ | 1,069.7023 |  |
| L57 | Taurolithocholic acid 3-glucuronide K^+^ | 698.2943 |  |
| L92 | PE-Cer(d15:2(4E,6E)/24:0(2OH)) Na^+^ | 767.5643 |  |
| L22 | PG(18:0/22:4(7Z,10Z,13Z,16Z)) K^+^ | 865.5285 |  |
| L72 | Fumonisin C4 Na^+^ | 698.3717 |  |
| L145 | (R)-(16,16-dimethyldocosa-cis-5,8,11,14-tetraenoyl)-1'-hydroxy-2'-propylamine K^+^ | 456.3273 |  |
| L54 | PI(P-20:0/17:2(9Z,12Z)) K^+^ | 899.5461 |  |
| L87 | PG(O-20:0/0:0) K^+^ | 565.3233 |  |
| L75 | 3-demethylubiquinone-9 Na^+^ | 803.5951 |  |
| L141 | PA(22:1(11Z)/0:0) Na^+^ | 515.3088 |  |
| L130 | PC(20:5(5Z,8Z,11Z,14Z,17Z)/0:0) K^+^ | 580.2847 |  |
| L59 | PG(13:0/18:4(6Z,9Z,12Z,15Z)) Na^+^ | 723.4230 |  |
| L7 | PG(O-18:0/15:0) Na^+^ | 745.5350 |  |
| L127 | NeuAcalpha2-3Galbeta-Cer(d18:1/16:0) Na^+^ | 1,013.6424 |  |
| L73 | PE(18:1(11Z)/22:6(4Z,7Z,10Z,13Z,16Z,19Z)) Na^+^ | 812.5139 |  |
| L11 | PA(22:2(13Z,16Z)/22:6(4Z,7Z,10Z,13Z,16Z,19Z)) H^+^ | 801.5417 |  |
| L66 | C18 Sulfatide K^+^ | 846.5160 |  |
| L44 | PS(16:0/18:1(9Z)) K^+^ | 800.4768 |  |
| L18 | PI(12:0/19:0) H^+^ | 797.5201 |  |
| L32 | PS(13:0/22:2(13Z,16Z)) H^+^ | 774.5225 |  |
| L34 | PA(O-18:0/21:0) Na^+^ | 755.5881 |  |
| L9 | PA(O-18:0/21:0) K^+^ | 771.5602 |  |
| L29 | 16:3-Glc-Cholesterol K^+^ | 819.5516 |  |
| L41 | PS(O-18:0/22:6(4Z,7Z,10Z,13Z,16Z,19Z)) H^+^ | 822.5654 |  |
| L25 | PS(P-16:0/22:6(4Z,7Z,10Z,13Z,16Z,19Z)) H^+^ | 792.5230 |  |
| L53 | PA(O-18:0/21:0) H^+^ | 733.5508 |  |
| L112 | PC(16:0/3:1(2E)) K^+^ | 588.3095 |  |
| L39 | PG(22:0/20:0) K^+^ | 901.6267 |  |
| L17 | PI(18:4(6Z,9Z,12Z,15Z)/22:4(7Z,10Z,13Z,16Z)) Na^+^ | 929.5089 |  |
| L33 | PA(O-16:0/22:4(7Z,10Z,13Z,16Z)) H^+^ | 711.5331 |  |
| L126 | Desmosine H^+^ | 526.2861 |  |
| L139 | Glycocholic acid 3-glucuronide K^+^ | 682.3205 |  |
| L82 | LysoPE(0:0/18:1(11Z)) Na^+^ | 502.2872 |  |
| L150 | 16:2-Glc-Cholesterol K^+^ | 821.5663 |  |
| L140 | PG(O-18:0/0:0) H^+^ | 499.3434 |  |
| L65 | Cholest-5-ene H^+^ | 371.3680 |  |
| L149 | N-docosahexaenoyl glutamic acid Na^+^ | 480.2728 |  |
| L134 | PS(O-20:0/14:0) Na^+^ | 772.5398 |  |
| L24 | Pelargonidin 3-(6''-caffeylglucoside) K^+^ | 633.0987 |  |
| L58 | PA(18:0/22:1(11Z)) K^+^ | 797.5457 |  |
| L10 | PS(15:1(9Z)/22:4(7Z,10Z,13Z,16Z)) H^+^ | 796.5170 |  |
| L86 | PI(12:0/21:0) H^+^ | 825.5505 |  |
| L81 | MGDG(18:3(9Z,12Z,15Z)/18:4(6Z,9Z,12Z,15Z)) H^+^ | 773.5202 |  |
| L6 | PA(O-20:0/17:2(9Z,12Z)) K^+^ | 739.5075 |  |
| L47 | Ubiquinol K^+^ | 769.5521 |  |
| L43 | PS(O-16:0/20:2(11Z,14Z)) Na^+^ | 796.5470 |  |
| L52 | PS(15:0/20:3(8Z,11Z,14Z)) H^+^ | 772.5167 |  |
| L50 | C16-OH Sulfatide H^+^ | 796.5184 |  |
| L70 | PS(17:1(9Z)/22:4(7Z,10Z,13Z,16Z)) H^+^ | 824.5447 |  |
| L5 | PS(13:0/22:0) Na^+^ | 800.5362 |  |
| L133 | PC(20:4(5Z,8Z,11Z,14Z)/0:0) Na^+^ | 566.3164 |  |
| L93 | LysoPC(20:3(5Z,8Z,11Z)) Na^+^ | 568.3325 |  |
| L19 | DG(20:5(5Z,8Z,11Z,14Z,17Z)/21:0/0:0)[iso2] K^+^ | 723.5334 |  |
| L60 | LacCer(d18:1/14:0) Na^+^ | 856.5724 |  |
| L76 | C20 Sulfatide K^+^ | 874.5436 |  |
| L100 | PI(22:0/22:2(13Z,16Z)) K^+^ | 1,013.6424 |  |
| L15 | PS(17:0/22:4(7Z,10Z,13Z,16Z)) H^+^ | 826.5599 |  |
| L80 | LysoPC(18:2(9Z,12Z)) Na^+^ | 542.3167 |  |
| L4 | DG(20:4(5Z,8Z,11Z,14Z)/21:0/0:0)[iso2] K^+^ | 725.5492 |  |
| L143 | 1α,25-dihydroxy-24a,24b,24c-trihomovitamin D3 K^+^ | 497.3384 |  |
| L8 | PA(O-18:0/19:1(9Z)) K^+^ | 741.5230 |  |

^1^Lipids are listed in the order (top to bottom) of appearance in Fig. 2D.
