# Supplementary material for: Metabolomics analyses identify platelet activating factors and heme breakdown products as Lassa fever biomarkers
Source: PLoS Negl Trop Dis. 2017 Sep 18;11(9):e0005943. doi: 10.1371/journal.pntd.0005943 (PMC5619842; doi:10.1371/journal.pntd.0005943)
Supplement: S7 Table — (DOCX) [file pntd.0005943.s007.docx]

S7 Table. Diagnostic sensitivity and specificity of top biomarkers comparing Lassa fever (fatal plus nonfatal) versus postLassa (acute plus nonacute) patients.

| **Identifier** | **Biomarker** | **ROC** | **Sensitivity** | **Specificity** |
| --- | --- | --- | --- | --- |
| PAF7 | PC(O-18:2(9Z,12Z)/2:0) H^+^ | 0.9 | 0.88 | 0.88 |
| PAF12 | PC(O-14:0/2:0) Na^+^ | 0.71 | 0.75 | 0.78 |
| PAF13 | PC(O-15:0/2:0) H^+^ | 0.72 | 0.75 | 0.63 |
| PAF4 | PC(O-16:1(11Z)/2:0) Na^+^ | 0.69 | 0.73 | 0.73 |
| PAF6 | PC(O-18:1(10E)/2:0) Na^+^ | 0.84 | 0.7 | 0.83 |
| PAF23 | Lyso-PAF C-18 H^+^ | 0.81 | 0.7 | 0.7 |
| M5 | Unknown 2 | 0.84 | 0.7 | 0.65 |
| PAF9 | PC(O-12:0/2:0) H^+^ | 0.76 | 0.65 | 0.65 |
| M18 | 1-Methylinosine Na^+^ | 0.75 | 0.63 | 0.73 |
| PAF24 | Lyso-PAF C-18 Na^+^ | 0.77 | 0.63 | 0.7 |
| PAF22 | PAF C-18 Na^+^ | 0.61 | 0.6 | 0.6 |
| PAF20 | Arachidonoyl PAF C-16 Na^+^ | 0.75 | 0.58 | 0.8 |
| M9 | Mesobilirubinogen H^+^ | 0.73 | 0.58 | 0.68 |
| PAF1 | PC(O-10:1(9E)/2:0) H^+^ | 0.61 | 0.58 | 0.65 |
| PAF17 | Lyso-PAF C-16 H^+^ | 0.86 | 0.58 | 0.63 |
| M17 | 1-Methylinosine H^+^ | 0.71 | 0.58 | 0.58 |
| M10 | Mesobilirubinogen Na^+^ | 0.53 | 0.58 | 0.58 |
| M4 | FMBP | 0.64 | 0.58 | 0.55 |
| PAF21 | PAF C-18 H^+^ | 0.81 | 0.55 | 0.75 |
| PAF5 | PC(O-18:1(10E)/2:0) H^+^ | 0.66 | 0.55 | 0.68 |
| PAF10 | PC(O-12:0/2:0) Na^+^ | 0.6 | 0.55 | 0.55 |
| PAF18 | Lyso-PAF C-16 Na^+^ | 0.55 | 0.53 | 0.6 |
| M12 | D-Urobilinogen/I-Urobilin Na^+^ | 0.49 | 0.53 | 0.45 |
| PAF8 | PC(O-18:2(9Z,12Z)/2:0) Na^+^ | 0.48 | 0.5 | 0.53 |
| PAF16 | PAF C-16 Na^+^ | 0.665 | 0.5 | 0.43 |
| PAF2 | PC(O-10:1(9E)/2:0) Na^+^ | 0.53 | 0.5 | 0.43 |
| PAF3 | PC(O-16:1(11Z)/2:0) H^+^ | 0.52 | 0.48 | 0.58 |
| PAF15 | PAF C-16 H^+^ | 0.43 | 0.48 | 0.45 |
| PAF14 | PC(O-15:0/2:0) Na^+^ | 0.44 | 0.48 | 0.4 |
| M11 | D-Urobilinogen/I-Urobilin H^+^ | 0.39 | 0.48 | 0.4 |
| PAF11 | PC(O-14:0/2:0) H^+^ | 0.53 | 0.45 | 0.48 |
| PAF19 | Arachidonoyl PAF C-16 H^+^ | 0.47 | 0.35 | 0.45 |
