# Supplementary figures and images for: Metabolomics analyses identify platelet activating factors and heme breakdown products as Lassa fever biomarkers
Source: PLoS Negl Trop Dis. 2017 Sep 18;11(9):e0005943. doi: 10.1371/journal.pntd.0005943 (PMC5619842; doi:10.1371/journal.pntd.0005943)

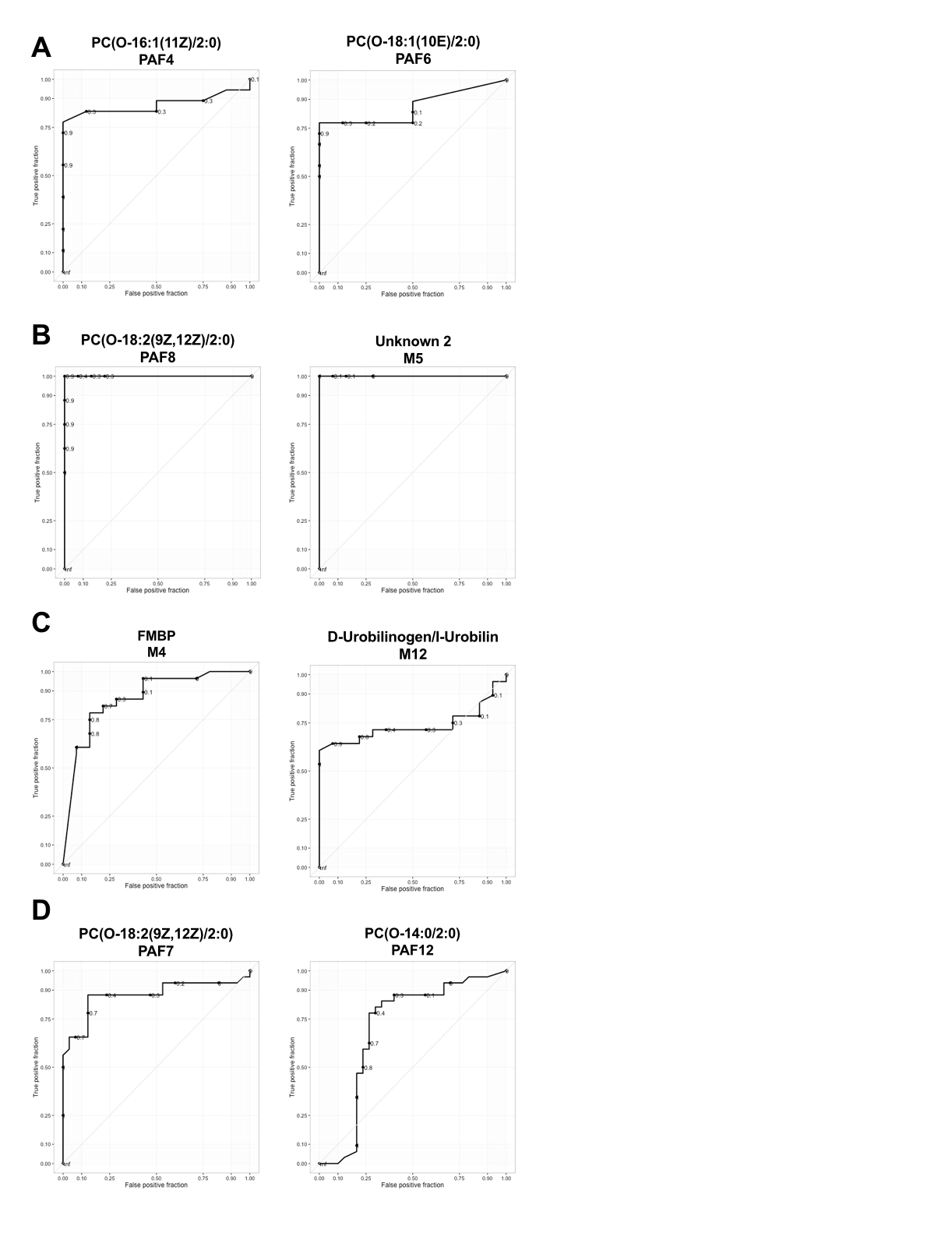

Supplement: S1 Fig — Panel A: fatal Lassa fever versus non-Lassa febrile illnesses. Panel B: fatal versus non-Lassa febrile illness. Panel C: acute Lassa fever (fatal plus non-fatal) versus non-Lassa febrile illnesses. Panel D: acute Lassa fever (fatal plus non-fatal) versus post Lassa febrile illnesses (Acute plus non-acute). (TIFF) [file pntd.0005943.s008.tiff]
